# Social isolation during the COVID-19 pandemic is associated with the decline in cognitive functioning in young adults

**DOI:** 10.7717/peerj.16532

**Published:** 2023-12-08

**Authors:** Ghulam Murtaza, Razia Sultana, Turki Abualait, Mishal Fatima, Shahid Bashir

**Affiliations:** 1 Department of Zoology, University of Gujrat, Gujrat, Punjab, Pakistan; 2 Department of Food Sciences, University of the Punjab, Lahore, Punjab, Pakistan; 3 College of Applied Medical Sciences, Imam Abdulrahman Bin Faisal University, Dammam, Eastern Province, Saudi Arabia; 4 Neuroscience Center, King Fahad Specialist Hospital, Dammam, Saudi Arabia

**Keywords:** COVID-19, Lockdown, Cognitive functions, Social isolation, Young adults

## Abstract

Coronaviruses have caused widespread disease and death worldwide, leading to the implementation of lockdown measures and the closure of educational institutions in various countries. This research aims to investigate the impact of social isolation on the cognitive functioning of young students. The study included 84 subjects, with 48 being socially isolated and 36 non-isolated individuals. The participants’ mental health was assessed using the Mini-Mental State Examination (MMSE), while cognitive functions were evaluated through attention-switching tasks (AST), pattern recognition memory (PRM), and choice reaction time (CRT) tests utilizing the Cambridge Neuropsychological Automated Battery (CANTAB) software. The socially isolated group had an average age of 21.3 ± 1.1 years, whereas the non-isolated group had an average age of 22.8 ± 2.0 years. The MMSE scores were 25.8 ± 1.6 for the socially isolated group and 28.6 ± 1.3 for the non-isolated group. In terms of cognitive functioning, there were significant differences (*p* = 0.000) observed in the values of AST correct latency for non-switching blocks (blocks 3 and 5) between the socially isolated group (608.1 ± 139.2) and the non-isolated group (499.5 ± 67.8). Similarly, the AST mean correct latency for switching blocks (block 7) was significantly different (*p* = 0.012) between the socially isolated group (784.4 ± 212.5) and the non-isolated group (671.8 ± 175.6). The socially isolated group exhibited significantly higher values in AST correct mean latency, AST congruent mean latency, AST incongruent mean latency, and AST percent mean correct trials compared to the non-isolated group. Additionally, the PRM mean percent correct significantly differed (*p* = 0.000) between the isolated group (81.3 ± 12.0) and the non-isolated group (91.9 ± 9.2). The isolated group also showed a higher CRT correct mean latency (482.4 ± 128.9) than the non-isolated group (451.0 ± 59.0), however the difference was not significant. In conclusion, social isolation during the COVID-19 pandemic has resulted in a decline in the cognitive functioning of young students.

## Introduction

A coronavirus is a large, enveloped, single-stranded RNA virus that can infect many animals and humans. The first documented coronavirus case was reported by Tyrrell & Bynoe (1966) when they successfully cultivated the viruses from a patient with the common cold. The seventh known human coronavirus, known as SARS-CoV-2, was initially identified in December 2019 in Wuhan, China, where it caused an acute respiratory syndrome *(*Zhou et al., 2020). As a precautionary measure, the authorities in Wuhan implemented a city-wide lockdown on January 23, 2020, in an attempt to contain the spread of the disease (Xiang et al., 2020). However, within a few months, COVID-19 cases emerged in numerous other countries, rapidly turning the disease into a global threat (Spina et al., 2020). The coronavirus has led to millions of infections and deaths worldwide. To mitigate its impact, many countries implemented lockdown measures, including the closure of educational institutions. Despite the lockdown, colleges and universities adapted by transitioning from face-to-face teaching to distance education, utilizing telematics and online resources (Fernandez Cruz et al., 2020).

Isolation or home confinement is an unsettling and unfamiliar experience that disrupts daily routines and separates individuals from their family and friends (Perrin et al., 2009). Human beings have an inherent need for social interaction, as it affects their well-being and helps them cope with stress and depression (Sherman, Kim & Taylor, 2009). Social support encompasses an interpersonal system involving care, love, respect, value, networking, communication, and more (Cohen & Wills, 1985). Strong social support has been shown to reduce difficulties in making career-related decisions (Li, 2014). Additionally, cultural symbols of wealth and social dominance activate reward-related neural pathways (Erk et al., 2002). Social exclusion can negatively affect an individual’s behavior and cognition (Niu et al., 2022). Research has demonstrated that social isolation, loneliness, and confinement impact physical health and mood (Lippi et al., 2020; Zhang et al., 2020). Poor social networks, loneliness, and a lack of close relationships increase the risk of dementia (Fratiglioni et al., 2000). Engaging in social networking, activities, and recreational pursuits also contribute to enhanced memory (Richards, Hardy & Wadsworth, 2003) and protection against cognitive decline (Bassuk, Glass & Berkman, 1999). Consequently, online teaching systems may have various psychological consequences.

The COVID-19 pandemic has created a public health emergency, highlighting the need for a comprehensive understanding of its impact on cognitive function. Cognitive reserve is often associated with the relationship between cognitive function and social isolation (Stern, 2009). As social networks and interactions provide brain stimulation, it is plausible that frequent social interaction can enhance or protect cognitive function (van Gelder et al., 2006). However, there is limited research on the cognitive changes experienced by young adults as a result of social isolation during the COVID-19 pandemic. It is crucial to investigate whether the lockdown conditions have led to cognitive decline, cognitive improvement, or no discernible effects on cognition. Such findings could have implications for educational concepts, daily life, and theories of cognitive decline (Ingram, Hand & Maciejewski, 2021*)*. This study explores the impact of social isolation during the COVID-19 lockdown on the cognitive functioning of young university students.

## Materials and Methods

### Selection of participants

In this cross-sectional study, participants were recruited and divided into two groups: a socially isolated group and a non-isolated group. Subjects in the isolated group had not attended university since the first lockdown on March 13, 2020. Their recruitment took place in December 2021, after 20.5 months of lockdown and shortly after the start of university classes on November 29, 2021. During the COVID-19 lockdown, they remained indoors at all times and were not allowed to leave without permission. They attended classes online while staying at home.

Subjects in the non-isolated group were recruited before the COVID-19 pandemic. Their demographic and cognitive data were obtained from our previous study conducted before the pandemic, as non-isolated subjects were unavailable for this study due to the nationwide lockdown conditions. For more details, please refer to Riaz et al. (2021).

Both groups of subjects were living with their families before and during the COVID-19 pandemic. All research participants were provided with an explanation of the study objectives and informed about their voluntary participation. Additionally, written informed consent was obtained from them for their involvement in the study. A questionnaire (Table 1) collected demographic information, including gender, age, marital status, and education. Forty-eight subjects were included in the socially isolated group, while the non-isolated group consisted of 36 subjects. Both groups contained male and single participants who were living with their families.

**Table 1:** Demographic data of research participants.

Parameters		Socially isolated group	Non-isolated group
Gender	Male (No.)	48	36
Age (years)	Mean ± SD	21.3 ± 1.1	22.8 ± 2.0
Education	Years of education	14	14–16
Marital status	Single (No.)	48	36
MMSE score	Mean ± SD	25.8 ± 1.6	28.6 ± 1.3
EHI score	Mean ± SD	99.6 ± 1.1	100 ± 0.0

**Note:**

MMSE, mini mental state examination; EHI, Edinburg handedness inventory; SD, standard deviation.

The sample size was determined based on previous studies (Bashir et al., 2017, 2020; Riaz et al., 2021). Consequently, a total of 48 socially isolated subjects were recruited, which was deemed sufficient to examine the association between social isolation and cognitive function.

### Exclusion standards

Subjects were interviewed about their daily routines and medical histories. Those found to be addicted to alcohol, smoking, or any other form of drugs, as well as individuals suffering from psychiatric disorders, musculoskeletal syndromes, seizures, vision impairments, malignancies, disrupted sleep patterns, diabetes, anemia, and obstructive lung disorders were excluded from the study (Bashir et al., 2017; Bashir et al., 2020).

### Ethical approval

The procedures of this study adhered to the standards set by the local Institutional Review Board (IRB) at the University of Gujrat. Ethical approval from the IRB was obtained prior to the commencement of the study (Ref: UOG/ORIC/2022/004).

### Cognitive function

The Mini-Mental State Examination (MMSE) (Folstein, Folstein & McHugh, 1975) and the Edinburgh Handedness Inventory (EHI) (Veale, 2014) were employed to assess the participants’ mental health and dominant hand, respectively. The authors obtained permission from the copyright holders to utilize the MMSE and EHI.

To investigate the association between social isolation and cognitive decline or enhancement, the researchers conducted tests using the Cambridge Neuropsychological Test Automated Battery (CANTAB) Research Suite Software. This software is a standard tool used for assessing cognitive functions such as attention, memory, and decision-making abilities (Chamberlain et al., 2012; Karlsen et al., 2022). As a computer-based assessment, CANTAB requires less time to complete. Moreover, the tests conducted using CANTAB are considered more accurate and reliable than article-based assessments (Morrison et al., 2015).

Before each test, participants were given a practice session to familiarize themselves with the test and assess their performance. The participants were comfortably seated in front of a computer, and response buttons were utilized during the tests. The entire testing process took approximately 25–30 min to complete.

### Attention-switching task (AST)

The attention-switching task (AST) evaluates a participant’s capacity to shift attention between the location and direction of an arrow displayed on a screen while disregarding irrelevant distractors that appear as attention-diverting events. It serves as a measure of executive function and assesses cognitive control processes associated with the prefrontal cortex.

During each trial of the AST, a cue is presented at the top of the screen, indicating to participants whether they should press the left or right button based on the location or direction of an arrow displayed on the screen. The AST yields results in the form of error scores and response latencies, capturing the accuracy and speed of participants’ responses.

### Pattern recognition memory (PRM)

The pattern recognition memory (PRM) test is a visual task that employs a paradigm to present visual patterns on a screen for participants to memorize. These patterns are challenging to label verbally. Subsequently, the patterns are presented again in pairs, and participants are required to identify the exact pattern they had previously memorized. The accuracy of their responses is measured in terms of the number of correct answers and the corresponding percentages.

### Choice reaction time (CRT)

This task assesses the participant’s decision-making speed and reaction time to stimuli. The stimuli consist of two choices represented by arrows displayed on either the right or left side of the screen. The reaction time (measured in milliseconds) is recorded when the participant presses the corresponding right or left button. The test evaluates both correct and incorrect responses, as well as the latency or speed of the participant’s reaction.

### Statistical analysis

The acquired data were analyzed using the SPSS software for statistical analysis. A comparison was made between the data of socially isolated subjects who attended classes online during the COVID-19 lockdown and those who were not socially isolated. To compare these two distinct groups, an independent t-test was employed, and significance was determined. Results were deemed significant for *p*-values ≤ 0.050. The standard deviation (SD) and mean difference were also calculated and compared.

## Results

The average ages of the socially isolated group and the non-isolated group were 21.3 ± 1.1 years and 22.8 ± 2.0 years, respectively. Subjects in the socially isolated group were pursuing their education after completing 14 years of schooling, whereas subjects in the non-isolated group had completed 14–16 years of education. A MMSE score of ≥24 out of 30 indicates mental stability. The MMSE scores for the socially isolated and non-isolated groups were 25.8 ± 1.6 and 28.6 ± 1.3, respectively. Handedness was determined using the EHI to ensure participants’ dominant hands were known for completing all the tests. The EHI scores for the socially isolated and non-isolated groups were 99.6 ± 1.1 and 100 ± 0.0, respectively (Table 1). Both groups were similar (without significant differences) in the parameters shown in Table 1.

The AST congruency cost (mean correct) was higher in the socially isolated group compared to the non-isolated group, while the AST switching cost (mean correct) was higher in the non-isolated group than in the socially isolated group, but these differences were not statistically significant (*p* = 0.396, *p* = 0.916, respectively). However, the AST correct latency (non-switching blocks; blocks 3 and 5) was significantly higher in the socially isolated group (608.1 ± 139.2) than in the non-isolated group (499.5 ± 67.8; *p* = 0.000). Similarly, the AST mean correct latency (switching blocks; block 7) was significantly higher in the socially isolated group (784.4 ± 212.5) compared to the non-isolated group (671.8 ± 175.6; *p* = 0.012). The AST correct mean latency, AST congruent mean latency, AST incongruent mean latency, and AST percent mean correct trials were all significantly higher in the socially isolated group (697.1 ± 156.5, 665.8 ± 166.7, 741.2 ± 158.4, and 80.2 ± 13.2, respectively) compared to the non-isolated group (584.0 ± 115.1, 554.0 ± 111.5, 616.3 ± 120.9, and 96.6 ± 2.3, respectively). The PRM mean percent correct was significantly different between the isolated group (81.3 ± 12.0) and the non-isolated group (91.9 ± 9.2; *p* = 0.000). The CRT correct mean latency was higher in the isolated group (482.4 ± 128.9) than in the non-isolated group (451.0 ± 59.0) but without significant difference (*p* = 0.179). Moreover, the difference in CRT percent correct trials between the two groups was also not significant (*p* = 0.652; Table 2).

**Table 2:** Comparison of cognitive functions (AST, PRM, and CRT) between socially isolated males and females.

Tests	Socially isolated group (Mean ± SD)	Non-isolated group (Mean ± SD)	*p*-value
AST-congruency cost	75.4 ± 87.3	62.3 ± 31.5	0.396
AST-latency (blocks 3, 5) (non-switching blocks)	608.1 ± 139.2	499.5 ± 67.8	0.000
AST-latency (block 7) (switching block)	784.4 ± 212.5	671.8 ± 175.6	0.012
AST-switching cost	176.3 ± 191.2	172.4 ± 127.7	0.916
AST-latency	697.1 ± 156.5	584.0 ± 115.1	0.000
AST-latency (congruent)	665.8 ± 166.7	554.0 ± 111.5	0.001
AST-latency (incongruent)	741.2 ± 158.4	616.3 ± 120.9	0.000
AST-percent correct trials	80.2 ± 13.2	96.6 ± 2.3	0.000
PRM-percent correct	81.3 ± 12.0	91.9 ± 9.2	0.000
CRT-latency	482.4 ± 128.9	451.0 ± 59.0	0.179
CRT-percent correct trials	97.8 ± 7.8	98.4 ± 1.8	0.652

**Note:**

AST, attention switching task; PRM, pattern recognition memory; CRT, choice reaction time; SD, standard deviation. PRM-Percent correct are the number of correct responses expressed as a percentage significance among groups was calculated by applying t -test for independence. *p*-values ≤ 0.05 were taken as statistically significant.

## Discussion

The present study aimed to assess the impact of social isolation on attention, memory, and CRT in young adult university students. The results revealed a decline in cognitive functioning among socially isolated participants. Notably, these findings align with recent studies, indicating that the COVID-19 pandemic-related lockdown and subsequent social isolation have significantly affected cognitive functions, specifically attention and memory, in healthy young adults (Bland et al., 2022; Ingram, Hand & Maciejewski, 2021).

Previous studies conducted before the COVID-19 pandemic have consistently demonstrated a relationship between social isolation and cognitive functioning. For instance, in a study by Wilson et al. (2007), 823 older adults (mean age 80.7 ± 7.1 years) without dementia were recruited to investigate various cognitive domains. Results showed that loneliness was associated with cognitive decline in multiple domains, including global cognition, semantic memory, episodic memory, working memory, visuospatial ability, and perceptual speed. Isolated individuals exhibited higher levels of anger, anxiety, negativity, and lower levels of optimism and security compared to those with strong social connections. Similarly, a study conducted by Lara et al. (2019) in Spain before the COVID-19 pandemic analyzed data from 1,691 participants aged 50 years or older to explore the impact of loneliness and social isolation on cognitive function. The findings revealed a significant association between social isolation and cognitive decline, as loneliness was linked to lower scores in composite cognitive measures, verbal fluency, immediate and delayed recall, and backward digit span. Furthermore, a study conducted in Appalachia prior to the pandemic examined data from 267 older adults aged 70 to 94 years. The study explored the relationship between cognition, social isolation, and loneliness. The results indicated a significant association between cognitive impairment and social isolation (DiNapoli, Wu & Scogin, 2014). Our study findings are consistent with the previous research; however, it is important to note that our study focused on a sample of young university students.

Previous studies examining the impact of social isolation on cognitive function during the COVID-19 pandemic have predominantly focused on older adults across a wide age range (Ingram, Hand & Maciejewski, 2021) or individuals with existing cognitive impairments such as dementia and Alzheimer’s disease (Manca et al., 2022). For instance, a recent study conducted in the US evaluated the mental health and cognition of adults aged 55 years and older over a 9-month period during the pandemic. The findings suggested that increased loneliness and social isolation had detrimental effects on cognitive perception (Kobayashi et al., 2022). Similarly, a study conducted in the UK by Bland et al. (2022) collected data from 92 participants aged 19 to 64 years through questionnaires to investigate the impact of the COVID-19 lockdown on emotional and social cognition. The study revealed significant negative effects of social isolation on participants’ cognitive processes.

Ingram, Hand & Maciejewski (2021) conducted a study involving 342 Scottish nationals, examining the effects of COVID-19 isolation on cognitive abilities in older adults ranging from 18 to 72 years. The results indicated that cognitive impairments were associated with individual experiences of social isolation (Ingram, Hand & Maciejewski, 2021). Furthermore, irrespective of age, lack of social communication during the COVID-19 lockdown was found to harm cognitive performance among Germans (Menze et al., 2022). Consistent with these previous studies conducted during the COVID-19 pandemic, our study observed a significant decline in cognitive functioning among young university students. Therefore, our findings align with the existing literature, highlighting the adverse effects of social isolation on cognitive function. By investigating these effects in a healthy young adult population, our study aimed to elucidate the influence of social isolation on cognitive functions while controlling for age-related changes in brain structure and function that may confound the results.

The theoretical mechanism underlying the relationship between social isolation and cognition remains largely unknown. However, previous research has provided some insights. For instance, a study by Cacioppo et al. (2011) demonstrated that chronic social isolation, social-threat hypervigilance, and neurobiological mechanisms may contribute to cognitive load and disruption of cognitive abilities. During the COVID-19 pandemic, it has been suggested that social isolation could impact cognitive functions due to limited opportunities for practicing cognitive skills or acquiring new coping methods to counteract cognitive decline. In a study involving individuals aged 75–85 years, cognition was assessed using the MMSE and clinical dementia rating at baseline. The study found associations between cognitive decline and factors such as the APOE4 genotype, elevated serum (ionized) calcium levels, and loneliness, each independently contributing to cognitive decline (Tilvis et al., 2004). Furthermore, chronic loneliness has been linked to elevated salivary cortisol levels, increased release of corticotropin-releasing hormone, and activation of the hypothalamic-pituitary-adrenocortical axis (Cacioppo et al., 2000). However, emotional support and appraisal/informational support have shown effectiveness in reducing negative emotions (Morese et al., 2019). Such support can also regulate neurobiological correlates and have positive neurophysiological effects. Moreover, it has been observed that support systems can reduce the risk of illness, facilitate recovery, and decrease mortality rates associated with various diseases (Sherman, Kim & Taylor, 2009).

This study has several limitations that should be acknowledged. First, the sample size was small, and the study design was cross-sectional. Additionally, data from the non-isolated participants were obtained from a previous study, as they could not be recruited for this specific investigation. The inability to include non-isolated participants in this study was due to the countrywide lockdown conditions, which led to the closure of educational institutions. Furthermore, information regarding the participants’ COVID-19 infection status was not collected. However, it should be noted that all subjects in the non-isolated group tested negative for COVID-19 during the previous study (Riaz et al., 2021), as COVID-19 was not prevalent in Pakistan at that time. Another limitation is that only male participants were included in the socially isolated group to compare and make the data homogenic with the non-isolated group. The data of non-isolated group were taken from our previous study (Riaz et al., 2021) in which recruitment of females for investigation was not possible due to cultural reasons.

Based on our findings, it is evident that the cognitive functioning of young students has been significantly affected by the COVID-19 pandemic. Therefore, further examination of this issue is crucial from a public health perspective. This study emphasizes the importance of future research that explores the correlation between neuroscience, psychology, decision-making, and the underlying neuroanatomical pathways. Such studies can provide valuable insights into the neurocognitive field. Additionally, it is necessary to conduct longitudinal studies with a larger sample size, employing multiple measures, to investigate social determinants such as social isolation and loneliness as potential negative predictors of cognitive function impairments. Furthermore, it would be valuable to explore social interventions that can contribute to the prevention and risk reduction of cognitive decline. These avenues of research have the potential to deepen our understanding of the topic and inform strategies for maintaining cognitive health.

## Supplemental Information

10.7717/peerj.16532/supp-1 Supplemental Information 1 Raw data.

## Additional Information and Declarations

### Data Availability

The following information was supplied regarding data availability: The data, from socially isolated and non-isolated groups of people, is available in the Supplemental File.
